# Influence of upper-body high-intensity intermittent training on energy metabolism and maximal oxygen uptake in elite swimmers

**DOI:** 10.3389/fphys.2025.1636405

**Published:** 2025-09-15

**Authors:** Lei Zhang, Hanyi Li, Tongling Wang, Chao Chen

**Affiliations:** ^1^ College of General Education, Wenzhou Business College, Wenzhou, China; ^2^ College of Physical Education, Dalian University, Dalian, China; ^3^ Institute of Physical Education, Huzhou University, Huzhou, China

**Keywords:** athletic performance, upper-body training, cardiopulmonary function, metabolic efficiency, high-intensity interval training (HIIT)

## Abstract

**Purpose:**

This paper aimed to investigate the effects of upper-body high-intensity interval training (HIIT) on energy metabolism and maximal oxygen uptake ( ${\text{VO}}_{2}$ max) in elite swimmers.

**Methods:**

A randomized controlled trial was conducted, in which elite swimmers were stratified and randomly assigned to either an upper-body HIIT group or an upper-body moderate-intensity continuous training (MICT) group. The HIIT group performed upper-body HIIT sessions lasting 60 min, including a warm-up, main workout, and cool-down at a 2:3:1 time ratio. The main workout consisted of circuit-based HIIT involving eight exercises, each performed for 20 s with 10 s of rest, totaling 230 s per circuit, with 3-min interset intervals, repeated for three sets. The MICT group followed a similar session structure except that the main workout involved eight continuous exercises performed for 60 s each with 20-s rest intervals and 20-s interset intervals and also repeated for three sets. Pre- and post-intervention assessments included upper-body cycle ergometry to evaluate the ${\text{VO}}_{2}$ max and indices of energy metabolism. Repeated-measure ANOVA was used to analyze changes in ${\text{VO}}_{2}$ max and energy metabolism indicators.

**Results:**

Prior to the intervention, no significant differences in ${\text{VO}}_{2}$ max or energy metabolism indices between the two groups were noted. After 4 weeks of training, the HIIT group exhibited significant improvements in ${\text{VO}}_{2}$ max and energy metabolism parameters as assessed by upper-body ergometry (p < 0.05). By contrast, the MICT group showed no significant changes in these indicators (p > 0.05). A significant interaction effect was observed between time and group (p < 0.05).

**Conclusion:**

A 4-week program of upper-body HIIT significantly enhances energy metabolism and ${\text{VO}}_{2}$ max in elite swimmers. These findings provide a theoretical basis for incorporating upper-body HIIT into the training regimens of competitive swimmers to optimize aerobic capacity and metabolic efficiency.

## 1 Introduction

High-intensity interval training (HIIT) is a modern exercise regimen characterized by alternating bouts of high-intensity activity and periods of passive or active recovery at moderate or low intensity Zhao et al. (2020); Coates et al. (2023). Compared with moderate-intensity continuous training (MICT), HIIT provides superior physiological stimuli due to its repeated bouts of high-intensity effort, which generate greater cardiovascular and metabolic stress, leading to improved ${\text{VO}}_{2}$ max, enhanced mitochondrial biogenesis, and increased excess postexercise oxygen consumption (EPOC), all contributing to better cardiopulmonary and metabolic adaptations Taylor et al. (2019). Moreover, HIIT reduces total training volume and time demands while mitigating the risks associated with early sport specialization Jayanthi et al. (2013); Atakan et al. (2021). In addition, HIIT improves cardiovascular fitness Bang-Kittilsen et al. (2022), muscular strength Merchant et al. (2021), athletic performance, and skeletal muscle energy metabolism Kistner et al. (2019).

Considering the unique demands of specific sports such as swimming is important. Swimming is a complex full-body activity that requires coordinated cyclic movements of the upper and lower limbs to overcome water resistance and to generate propulsion. The strength, endurance, and efficiency of the upper limbs play a critical role in determining swimming performance Barbosa et al. (2013); Guignard et al. (2019). Muscle strength is particularly important for sprint swimming Sharp et al. (1982), and upper-body strength and power output have been linked to maximal swim velocity over distances ranging from 25 m to 400 m Hawley and Williams (1991); Hawley et al. (1992). Therefore, upper-body training is a vital component of swimmers’ overall training regimens and directly influences speed and endurance. Emphasis on upper-limb conditioning is essential for optimizing swimming performance and technique Zwierzchowska et al. (2023).

Given the critical role of muscle function in swimming, understanding the underlying energy metabolism becomes essential. Energy metabolism is a fundamental physiological process required to sustain basic life functions Zhu et al. (2022), encompassing aerobic and anaerobic systems Latham et al. (2022). For endurance events and short, high-intensity efforts, understanding an athlete’s metabolic profile is essential for designing effective training programs Brooks and Mercier (1994). In swimming, events typically range from 22 s to 15 min (50–1500 m), and energy demands are primarily met through anaerobic and aerobic glycolytic pathways Hollander et al. (2005). Beyond performance enhancement, metabolic training influences body composition, energy efficiency, recovery, and injury prevention, which makes it a cornerstone of athletic conditioning across disciplines Bangsbo (1994); Mujika and Padilla (2001). Repeated HIIT sessions promote mitochondrial biogenesis in muscle cells and enhance ATP production via aerobic pathways Ryan et al. (2020); Cordeiro et al. (2021). HIIT also increases the activity of key enzymes involved in anaerobic glycolysis and improves the muscle’s capacity to generate energy through lactate metabolism during brief, high-intensity exertion Yt et al. (2019).

Closely related to energy metabolism is the concept of maximum oxygen uptake ( ${\text{VO}}_{2}$ max), which is another key factor for swimmers. ${\text{VO}}_{2}$ max is widely regarded as the gold standard for evaluating cardiorespiratory fitness and serves as a core indicator of athletic performance Bassett and Howley (2000); Kurl et al. (2022). For swimmers, an increase in ${\text{VO}}_{2}$ max reflects improved cardiovascular health and is linked to performance, particularly in middle- and long-distance events. This association arises from the enhanced capacity to sustain higher exercise intensity through more efficient oxygen utilization Truijens et al. (2003); Aspenes et al. (2009). Previous research confirmed that HIIT is a time-efficient, effective method for improving ${\text{VO}}_{2}$ max Wen et al. (2019); Gibala (2021). Therefore, incorporating HIIT into training may be beneficial for swimmers aiming to enhance ${\text{VO}}_{2}$ max and overall performance.

While evidence for HIIT’s benefits is extensive in various sports disciplines, its specific application to swimming warrants further exploration. Numerous studies have confirmed the effectiveness of HIIT in enhancing sport-specific performance, such as increasing punching power and endurance in boxing Davis et al. (2015), sprint capacity in kayaking Du and Tao (2022), and serve velocity in volleyball Sheppard et al. (2007). However, research specifically targeting swimmers remains limited. This gap gives rise to the current paper’s aim to investigate the influence of upper-body HIIT on energy metabolism and ${\text{VO}}_{2}$ max in elite swimmers. Existing literature on HIIT in swimming has primarily focused on whole-body training and its effects on cardiorespiratory endurance, and limited attention has been given to how upper-body HIIT alone may optimize energy utilization and performance outcomes. Therefore, this paper aims to address this research gap by providing empirical evidence on the effectiveness of upper-body HIIT in competitive swimming. Using a quantitative research design, ${\text{VO}}_{2}$ max and energy metabolism data are collected and analyzed before and after a targeted HIIT intervention to evaluate, with scientific rigor, the specific effects of upper-body HIIT on elite swimmers, extend the application of HIIT in aquatic sports, and offer athletes and coaches a potentially more time-efficient, performance-enhancing training strategy. Notably, this paper focuses on metabolic and cardiorespiratory adaptations to upper-body HIIT rather than direct measurements of in-water performance. While kinematic variables (e.g., stroke rate) are critical for swimming performance, this paper’s primary goal is to establish the physiological mechanisms underlying upper-body metabolic responses to targeted HIIT and to provide a foundation for future sport-specific investigations.

## 2 Research subjects and methods

### 2.1 Research subjects

*A priori* power analysis was conducted using G*Power 3.1.9.7 Faul et al. (2007) to determine the required sample size. For a 2 × two repeated-measure ANOVA with an alpha level of 0.05, an effect size of f = 0.4, and a desired power of 0.80, the minimum total sample size was estimated as 16 participants (8 per group). To account for potential attrition or data loss, the final sample size was set at 24 participants.

Twenty-four swimmers (16 males, 8 females) from local sports faculty were voluntarily recruited (see Table 1 for basic participant information) through official university announcements, coach recommendations, and on-campus information sessions to ensure they met the required training background and could consistently participate in the study. Inclusion and exclusion criteria were as follows: (1) Athletes must meet or exceed the national first-class athlete standard (Note: According to China’s 2025 Swimming Athlete Technical Grade Standards, first-class athletes must achieve benchmark times in official competitions, for example, 55.50 s for men’s 100 m freestyle [50 m pool] or 1:02.50 for women’s 100 m freestyle, verified via electronic timing). (2) Participants must be aged 18–24 years and free from chronic pain or cardiovascular disease. (3) Individuals with medical conditions contraindicating high-intensity exercise were excluded. (4) Those unable to train due to sports injuries were excluded. (5) Athletes below the required competitive level were excluded. Regarding sport specialization, all participants specialized in Olympic swimming events, and primary disciplines included freestyle (50, 100, or 400 m), backstroke (100 or 200 m), and butterfly (100 or 200 m), as confirmed via coach verification and competition records.

**TABLE 1:** Basic information of swimmers.

Indicator	Mean ± SD
Age (years)	20.71 ± 1.38
Height (cm)	178.16 ± 8.74
Weight (kg)	72.09 ± 13.01
Training Years	10.59 ± 2.62

The study was approved by the Local Ethics Committee (Approval Number 102772021RT031), and all participants provided written informed consent. To minimize external variables, participants avoided high-intensity training 24 h before testing, maintained regular dietary and sleep routines throughout the testing period, and consumed meals at least 2 h pretest (with moderate water intake permitted) to avoid fasting or postprandial states.

### 2.2 Study design

This study was a 4-week experimental longitudinal investigation designed to examine the specific effects of upper-body high-intensity interval training (HIIT) on energy metabolism and ${\text{VO}}_{2}$ max. By rigorously controlling intervention variables, the study aimed to isolate and evaluate the independent effects of upper-body HIIT, thereby clarifying its targeted impact on metabolic function and maximal oxygen uptake. The testing and intervention phases were conducted from November to December 2022 at the Strength and Conditioning Research Center of local sports faculty. All assessments were performed using an upper-body cycle ergometer (Lode Excalibur Sport, Lode BV, Groningen, Netherlands), with pre-tests (baseline measurements) completed within 7 days prior to the start of the 4-week intervention, and post-tests conducted within 7 days after the completion of the intervention. The pre- and post-tests were separated by a minimum interval of 4 weeks to ensure sufficient time for the intervention effect to manifest Ruddy and Carson (2013). To maintain consistency, each participant underwent testing at approximately the same time of day. Prior to all performance tests, anthropometric measurements—including height and body weight—were taken to assess baseline physical characteristics (Height was measured using a standard stadiometer, and body weight was assessed with a calibrated digital scale).

### 2.3 Testing protocol and intensity monitoring

#### 2.3.1 Equipment

The following equipment and materials were utilized throughout the study. An upper-body cycle ergometer was used to conduct incremental load testing. A portable metabolic analyzer (COSMED K5, Rome, Italy) was employed to measure respiratory gas exchange parameters during and after exercise. Heart rate was monitored in real time using a Polar heart rate strap (Polar Accurex Plus, Polar Electro Oy, Kempele, Finland). Blood lactate concentrations were assessed using a benchtop blood lactate analyzer (BIOSEN S Line, EKF Diagnostic, Barleben, Germany). Additional materials included the Borg Rating of Perceived Exertion (RPE) scale, sterile lancets, EKF blood sampling tubes, alcohol swabs, medical-grade rubber gloves, a stopwatch, and marker pens.

#### 2.3.2 Testing methods and indicators

##### 2.3.2.1 Gas exchange data collection

Respiratory gas exchange was continuously measured during all exercise tests using a portable metabolic cart (Cosmed Quark RMR, Rome, Italy), which was calibrated before each testing session using standard gases (16% ${\text{O}}_{2}$ and 5% ${\text{CO}}_{2}$ ) and ambient air. Participants wore a facemask connected to the metabolic cart, which recorded (1) ${\text{O}}_{2}$ consumption ( ${\text{VO}}_{2}$ , mL ⋅ ${\text{kg}}^{-1}\cdot$ ${\text{min}}^{-1}$ ), (2) ${\text{CO}}_{2}$ production ( ${\text{VCO}}_{2}$ , mL ⋅ ${\text{kg}}^{-1}$ ⋅ ${\text{min}}^{-1}$ ), and (3) ventilation (VE, L ⋅ ${\text{min}}^{-1}$ ). These data were used to calculate total aerobic energy contribution (via accumulated ${\text{VO}}_{2}$ ). Data were sampled at 10-s intervals and averaged over 30-s epochs to ensure stability.

##### 2.3.2.2 Validation and quality control

Equipment Accuracy: The metabolic cart was calibrated before each testing session for gas concentration and flowmeter precision.

Steady-State Requirement: Aerobic energy contribution was only calculated during exercise stages with stable ${\text{VO}}_{2}$ and ${\text{VCO}}_{2}$ (coefficient of variation < 5% over 2 min) to ensure reliable measurement of oxygen uptake.

EPOC was not used in energy system contribution calculations because its primary role is quantifying postexercise recovery energy expenditure.

#### 2.3.3 Ergometer setup

The height of the upper-body ergometer was adjusted individually to ensure standardized positioning for each participant. Specifically, when the elbow was fully extended, the crank axis was aligned with the midpoint of the forearm; when the elbow was flexed, the elbow joint remained at the same horizontal level as the axis. This positioning guaranteed consistency across all tests. The training load on the Lode upper-body ergometer was 3% of the participant’s body mass, following the protocol of Franchini et al. Franchini et al. (2016). Torque (T) was calculated using the formula:

T = body mass × 3% × 9.8 × crank radius, resulting in a load expressed in Newton-meters (N ⋅ m).

#### 2.3.4 Testing procedure

**FIGURE 1:**
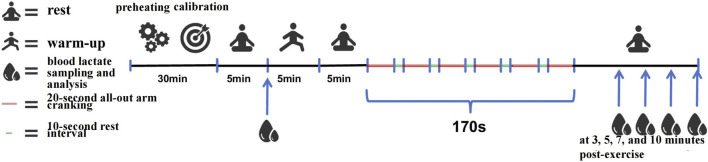
Schematic diagram of the test procedure.

Prior to testing, the K5 gas analysis system underwent a 30-min warm-up, followed by calibration procedures in accordance with the manufacturer’s specifications. Calibration included barometric pressure, gas concentration using standard calibration gas (comprising 15.00% ${\text{O}}_{2}$ , 5.00% ${\text{CO}}_{2}$ , and 80.00% ${\text{N}}_{2}$ ), and volume calibration using a 3-L calibration syringe. Gas collection, storage, and analysis were conducted using the manufacturer’s proprietary software (Mate Soft, COSMED, Italy). The overall testing procedure is illustrated in Figure 1. Upon arrival at the testing site, each athlete was instructed to remain seated quietly for 5 min to allow measurement of baseline (resting) blood lactate levels. This step was followed by a 5-min warm-up on a treadmill at a constant speed of 10 km/h. After the warm-up, participants rested for an additional 5 min, during which the previously calibrated K5 gas analyzer was fitted to the subject. The testing protocol consisted of six sets of maximal arm cranking for 20 s, each separated by 10-s rest intervals, for a total of 180 s of exercise. Blood lactate samples (20 µL) were collected from the earlobe at 3, 5, 7, and 10 min postexercise and examined using a lactate analyzer. Following the final sample collection at 10 min postexercise, the K5 device was removed. During this recovery period, athletes remained seated and were instructed to rest passively. Subjects were also advised to minimize verbal communication while wearing the K5 system to avoid data interference.

#### 2.3.5 Quality control

During each testing session, standardized movement techniques were strictly monitored to ensure performance consistency and data reliability. Technical deviations were corrected promptly to prevent injury, and verbal encouragement was provided to participants struggling with protocol completion. All tests followed the designated sequence and standardized rest intervals as per the experimental design.

### 2.4 Training protocol and intensity monitoring

#### 2.4.1 Training protocol

The training program lasted 4 weeks, with three sessions per week, totaling 12 sessions. Each session lasted 60 min and consisted of three parts: 20 min of warm-up, 30 min of upper-body HIIT or MICT, and 10 min of stretching and relaxation at the end.

The upper-body HIIT protocol was based on the Tabata model, which is widely recognized for its efficiency in stimulating aerobic and anaerobic energy systems through repeated short bursts of maximal effort followed by brief rest periods Tabata (2019); Tabata et al. (1996). Specifically, the HIIT sessions included eight resistance exercises targeting key upper-body muscle groups involved in swimming propulsion: resistance band incline pull-down, resistance band bent-over lateral pull, resistance band push-up, resistance band rear pull, resistance band front pull-down, resistance band prone pull-down, resistance band shoulder press, and decline push-up. Each exercise was performed for 20 s at maximum intensity, followed by a 10-s rest. This circuit lasted approximately 230 s and was repeated for three sets with a 3-min rest interval between sets, in line with protocols shown to improve ${\text{VO}}_{2}$ max and metabolic function effectively Franchini et al. (2016); Milanović et al. (2015); Helgerud et al. (2007).

Resistance bands were used as the main training equipment to provide progressive resistance across the full range of motion. This choice is supported by previous studies demonstrating that elastic resistance is a practical and effective method for improving muscle strength and endurance in both general fitness and sport-specific dry-land training for swimmers Aspenes et al. (2009); Hibbs et al. (2008). Moreover, studies comparing resistance bands with free weights and machines have shown comparable benefits for strength gains and functional performance (Colado et al., 2009; Andersen et al., 2010). A 25 kg yellow resistance band was used in all sessions to ensure adequate training load.

**FIGURE 2:**
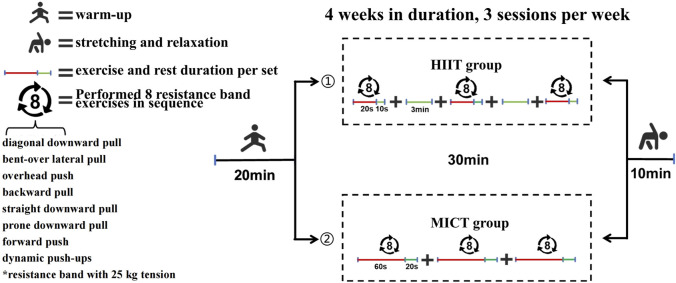
Schematic diagram of the training protocol.

The MICT group followed the same session duration, warm-up, and cool-down as the HIIT group but performed the main workout with moderate-intensity continuous resistance band exercises. Each exercise was performed continuously for 60 s with a 20-s rest, emphasizing endurance and aerobic metabolism. This design allows a direct comparison between upper-body HIIT and MICT on energy metabolism and ${\text{VO}}_{2}$ max improvements. Figure 2 illustrates the training protocol.

#### 2.4.2 Training monitoring

The HRmax estimation using the formula “208 - (0.7 × age)” was implemented to standardize training intensity monitoring across the intervention period and to align with ACSM guidelines for exercise prescription in healthy adults. While CPET provided direct ${\text{VO}}_{2}$ max measurements, continuous HRmax reassessment via CPET during daily training was impractical. The chosen intensity thresholds ( ≥ 85% HRmax for HIIT and 60%–69% HRmax for MICT) were based on ACSM’s classification of “vigorous intensity” (77%–95% HRmax) and “moderate intensity” (64%–76% HRmax), respectively, and ensured scientific validity for stimulating metabolic adaptations.

To enhance precision, training intensity was simultaneously monitored using Polar heart rate straps and the Borg RPE scale, per recommendations to combine objective and subjective measures when relying on estimated HRmax. This dual approach minimized errors from HRmax estimation variability ( ± five to eight bpm for the Gellish formula) and ensured participants maintained target intensities safely.

### 2.5 Statistical analysis

Data were tabulated and analyzed using the Statistical Package for the Social Sciences (SPSS, Version 25). Statistical significance was set at p<0.05 . Descriptive statistics were used to report means, standard deviations, and percentages. A 2 × two repeated measures analysis of variance (ANOVA) was conducted to examine the effects of group (HIIT vs. MICT) and time (pre-vs. post-intervention), reporting F -values, p -values, and partial eta-squared $\left(\eta_{p}^{2}\right)$ as the effect size measure to assess practical significance. Effect sizes were interpreted using the following thresholds: small effect $\left(\eta_{p}^{2}\approx 0.01\right)$ , medium effect $\left(\eta_{p}^{2}\approx 0.06\right)$ , and large effect $\left(\eta_{p}^{2}\approx 0.14\right)$ , providing a framework for evaluating the magnitude of observed effects. Graphical representations of results and trends were generated using Prism (Viewer Mode, Version 10) to facilitate visual interpretation.

## 3 Results and analysis

### 3.1 Participant characteristics

All participants completed the full testing and training intervention. The baseline characteristics of the athletes are presented in Table 2. There were no significant differences between groups in any of the measured variables, including age, height, body mass, and training years (p > 0.05).

**TABLE 2:** Basic information of swimmers.

Characteristics	Upper - body HIIT group	Upper - body MICT group	t	p
Age (years)	20.25 ± 1.23	21.17 ± 1.46	−1.67	0.11
Height (cm)	178.78 ± 9.22	177.54 ± 8.27	0.35	0.73
Weight (kg)	70.22 ± 13.54	73.95 ± 12.47	−0.70	0.49
Training experience	9.50 ± 2.47	11.67 ± 2.66	−2.07	0.5

### 3.2 The impact of upper-body high-intensity intermittent training on energy metabolism

A 2 (Group: Upper-Body HIIT vs. Upper-Body MICT) × 2 (Time: Pre-vs. Post-) repeated measures ANOVA was used to analyze glycolysis within energy metabolism, with results presented in Table 3. The analysis revealed a significant main effect of group, with the Upper-Body HIIT group exhibiting higher glycolysis levels than the Upper-Body MICT group ( F(1,22)=11.133 , p<0.01 , $\eta_{p}^{2}=0.336$ ). A significant main effect of time was also found, with higher glycolysis levels post-intervention than pre-intervention ( F(1,22)=70.383 , p<0.001 , $\eta_{p}^{2}=0.762$ ). Moreover, the interaction between groups and timing proved significant ( F(1,22)=87.882 , p<0.001 , $\eta_{p}^{2}=0.800$ ).

**TABLE 3:** Repeated measures ANOVA tests for glycolysis of different groups and test timing.

Group	Pre	Post	Group effect			Time effect			Interaction		
			F	P	η ^2^p	F	P	η ^2^p	F	P	η ^2^p
MICT	35.51 ± 9.19	34.66 ± 8.96									
			11.133	0.01	0.336	70.383	0.001	0.762	87.882	0.001	0.8
HIIT	39.12 ± 8.69	52.42 ± 8.47									

To delve deeper into the interaction between the group and timing, a simple effects analysis was conducted. For the Upper-Body HIIT group, pre-intervention glycolysis levels were significantly lower than post-intervention levels ( F(1,22)=157.780 , p<0.001 , $\eta_{p}^{2}=0.878$ ). Conversely, no significant difference between pre- and post-intervention glycolysis levels was observed in the Upper-Body MICT group ( F(1,22)=0.485 , p>0.05 , $\eta_{p}^{2}=0.022$ ). At the pre-intervention stage, glycolysis levels did not significantly differ between the two groups ( F(1,22)=0.976 , p>0.05 , $\eta_{p}^{2}=0.042$ ). However, at the post-intervention stage, glycolysis levels were significantly higher in the Upper-Body HIIT group than in the Upper-Body MICT group ( F(1,22)=30.807 , p<0.001 , $\eta_{p}^{2}=0.583$ ).

A 2 (Group: Upper-Body HIIT vs. Upper-Body MICT) × 2 (Time: Pre-vs. Post-) repeated-measures ANOVA was used to analyze the aerobic oxidation within energy metabolism, with results presented in Table 4. The analysis revealed a significant main effect of group ( F(1,22)=5.189 , p<0.05 , $\eta_{p}^{2}=0.191$ ), with the Upper-Body HIIT group exhibiting higher aerobic oxidation levels than the Upper-Body MICT group. A significant main effect of timing was also found ( F(1,22)=69.359 , p<0.001 , $\eta_{p}^{2}=0.759$ ), with higher aerobic oxidation levels post-intervention than pre-intervention. Moreover, the interaction between group and timing was significant ( F(1,22)=63.809 , p<0.001 , $\eta_{p}^{2}=0.744$ ).

**TABLE 4:** Repeated-measure ANOVA tests for aerobic oxidation of different groups and test timing.

Group	Pre	Post	Group effect			Time effect			Interaction		
			F	P	η ^2^p	F	P	η ^2^p	F	P	η ^2^p
MICT	102.45 ± 21.63	103.35 ± 19.83									
			5.189	0.05	0.191	69.359	0.001	0.759	63.809	0.001	0.744
HIIT	104.94 ± 30.83	148.17 ± 30.73									

To further explore the interaction effect between group and timing, simple effects analyses were conducted. For the Upper-Body HIIT group, pre-intervention aerobic oxidation levels were significantly lower than post-intervention levels ( F(1,22)=133.111 , p<0.001 , $\eta_{p}^{2}=0.858$ ). In contrast, no significant difference between pre- and post-intervention aerobic oxidation levels was observed in the Upper-Body MICT group ( F(1,22)=0.058 , p>0.05 , $\eta_{p}^{2}=0.003$ ). At the pre-intervention stage, aerobic oxidation levels did not significantly differ between the two groups ( F(1,22)=0.053 , p>0.05 , $\eta_{p}^{2}=0.002$ ). However, at the post-intervention stage, aerobic oxidation levels were significantly higher in the Upper-Body HIIT group than in the Upper-Body MICT group ( F(1,22)=18.017 , p<0.001 , $\eta_{p}^{2}=0.450$ ).

A 2 (Group: Upper-Body HIIT vs. Upper-Body MICT) × 2 (Time: Pre-vs. Post-) repeated measures ANOVA was conducted to analyze the phosphagen content in energy metabolism, with results presented in Table 5. The analysis revealed a significant main effect of group ( F(1,22)=6.376 , p<0.05 , $\eta_{p}^{2}=0.225$ ), with higher phosphagen content in the Upper-Body HIIT group than in the Upper-Body MICT group. A significant main effect of time was also found ( F(1,22)=25.342 , p<0.001 , $\eta_{p}^{2}=0.535$ ), with higher phosphagen content post-intervention than pre-intervention. Furthermore, the interaction between group and time proved significant ( F(1,22)=29.338 , p<0.001 , $\eta_{p}^{2}=0.571$ ).

**TABLE 5:** Repeated measures ANOVA tests for phosphagen of different groups and test timing.

Group	Pre	Post	Group effect			Time effect			Interaction		
			F	P	η ^2^p	F	P	η ^2^p	F	P	η ^2^p
MICT	37.19 ± 8.77	36.63 ± 8.12									
			6.376	0.05	0.225	25.342	0.001	0.535	29.338	0.001	0.571
HIIT	39.72 ± 9.54	55.17 ± 15.37									

To further explore the interaction between group and time, simple effects analyses were conducted. For the Upper-Body HIIT group, pre-intervention phosphagen content was significantly lower than post-intervention content ( F(1,22)=54.607 , p<0.001 , $\eta_{p}^{2}=0.713$ ). In contrast, no significant difference in phosphagen content between pre- and post-intervention was observed in the Upper-Body MICT group ( F(1,22)=0.073 , p>0.05 , $\eta_{p}^{2}=0.003$ ). At the pre-intervention stage, phosphagen content did not significantly differ between the two groups ( F(1,22)=0.455 , p>0.05 , $\eta_{p}^{2}=0.020$ ). However, at the post-intervention stage, phosphagen content was significantly higher in the Upper-Body HIIT group than in the Upper-Body MICT group ( F(1,22)=13.657 , p<0.01 , $\eta_{p}^{2}=0.383$ ).

### 3.3 The impact of upper-body high-intensity intermittent training on maximal oxygen uptake

A 2 (Group: Upper-Body HIIT vs. Upper-Body MICT) × 2 (Time: Pre-vs. Post-) repeated measures ANOVA was conducted to analyze maximal oxygen uptake ( ${\text{VO}}_{2}$ max), and the results are presented in Table 6. The analysis revealed a significant main effect of group ( F(1,22)=7.359 , p<0.05 , $\eta_{p}^{2}=0.251$ ), with the Upper-Body HIIT group showing higher ${\text{VO}}_{2}$ max levels than the Upper-Body MICT group. The main effect of timing was not significant ( F(1,22)=2.617 , p>0.05 , $\eta_{p}^{2}=0.106$ ), indicating no significant difference in ${\text{VO}}_{2}$ max between the pre- and post-tests overall. However, the correlation between group and timing was significant ( F(1,22)=18.904 , p<0.001 , $\eta_{p}^{2}=0.462$ ).

**TABLE 6:** Repeated-measures ANOVA tests of maximum oxygen uptake in different groups and test times.

Group	Pre	Post	Group effect			Time effect			Interaction		
			F	P	η ^2^p	F	P	η ^2^p	F	P	η ^2^p
Control	49.40 ± 3.75	48.19 ± 3.54									
			7.359	0.05	0.251	2.617	0.05	0.106	18.904	0.001	0.462
Upper	51.00 ± 2.92	53.63 ± 3.15									

To further explore the interaction between group and time, simple effects analyses were conducted. For the Upper-Body HIIT group, pre-test ${\text{VO}}_{2}$ max levels were significantly lower than post-test levels ( F(1,22)=17.794 , p<0.001 , $\eta_{p}^{2}=0.447$ ). In contrast, no significant difference between pre- and post-test ${\text{VO}}_{2}$ max levels was observed in the Upper-Body MICT group ( F(1,22)=3.727 , p>0.05 , $\eta_{p}^{2}=0.145$ ). At the pre-test stage, ${\text{VO}}_{2}$ max levels did not significantly differ between the two groups ( F(1,22)=1.353 , p>0.05 , $\eta_{p}^{2}=0.058$ ). However, at the post-test stage, ${\text{VO}}_{2}$ max levels were significantly higher in the Upper-Body HIIT group than in the Upper-Body MICT group ( F(1,22)=15.823 , p<0.01 , $\eta_{p}^{2}=0.418$ ).

**FIGURE 3:**
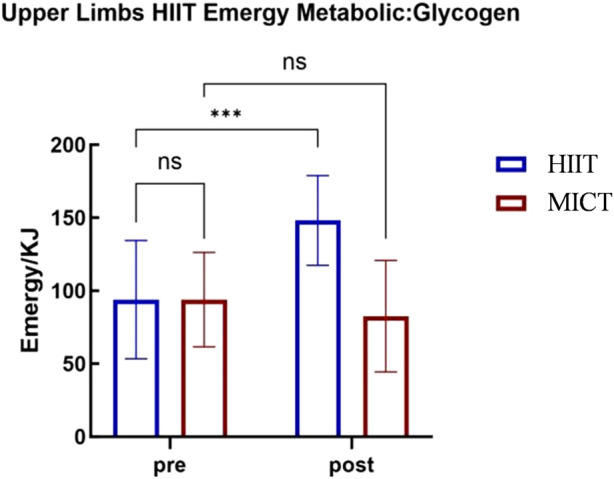
Upper-body cycle ergometry glycolysis energy supply characteristics.

**FIGURE 4:**
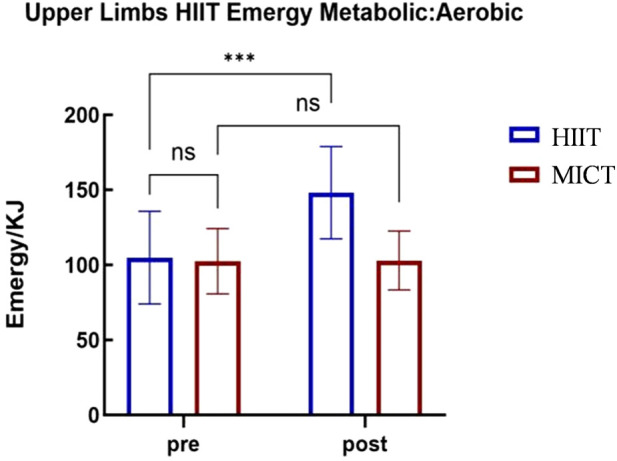
Upper-body cycle ergometry aerobic oxidation energy supply characteristics.

**FIGURE 5:**
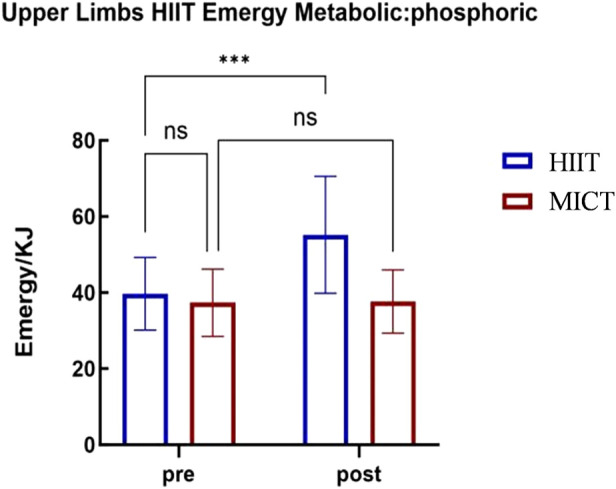
Upper-body cycle ergometry phosphagen energy supply characteristics.

**FIGURE 6:**
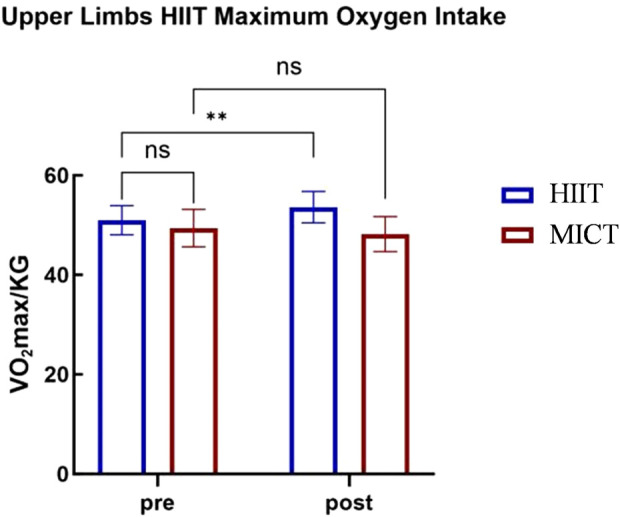
Upper-body cycle ergometry maximal oxygen uptake characteristics.

These findings indicate that 4 weeks of upper-body HIIT significantly enhances upper-body energy metabolism and ${\text{VO}}_{2}$ max. The 4-week upper-body HIIT intervention proved effective for improving these parameters. Analysis via upper-body cycle ergometry (see Figures 3–6) revealed no significant pre-intervention differences between the Upper-Body HIIT and MICT groups across the three energy systems and ${\text{VO}}_{2}$ max. However, post-intervention, the Upper-Body HIIT group demonstrated more efficient energy regulation and higher ${\text{VO}}_{2}$ max than the MICT group.

## 4 Discussion

This study demonstrated the significant impact of upper-body high-intensity interval training (HIIT) on enhancing energy metabolism and ${\text{VO}}_{2}$ max in swimmers. After 4 weeks of upper-body-focused HIIT, participants showed marked improvements in phosphagen, aerobic, and glycolytic energy systems, as well as ${\text{VO}}_{2}$ max. These results suggest that upper-body HIIT can be an effective method for improving swimming performance. The findings complement existing sports training literature and offer swim-specific insights for upper-limb strength and endurance development.

We speculate that these improvements stem from HIIT’s dual stimulation of the cardiovascular and muscular systems. According to Mitropoulos et al. (2018), short bursts of high-intensity effort in HIIT can enhance cardiac output and vascular function. Laursen and Jenkins Laursen and Jenkins (2002) proposed that HIIT improves ${\text{VO}}_{2}$ max by increasing stroke volume and blood volume, as well as by enhancing the muscles’ ability to extract oxygen. Dohlmann et al. (2018) also found that HIIT can promote mitochondrial density in muscle, thereby improving energy metabolic pathways and muscular energy efficiency. These adaptations are particularly crucial for swimmers, whose performance largely relies on upper-body strength and endurance to sustain repetitive movement patterns.

The training protocol employed in this study was based on a 20-s work and 10-s rest interval format, closely aligned with the Tabata training model. Tabata is widely recognized as one of the most effective forms of HIIT owing to its capacity to enhance aerobic and anaerobic energy systems simultaneously Tabata (2019). Previous studies have consistently reported improvements in cardiorespiratory fitness following HIIT, as assessed by ${\text{VO}}_{2}$ max Kessler et al. (2012); Lu et al. (2023). Specifically, these investigations observed an average increase of 12.87% ± 7.16% in ${\text{VO}}_{2}$ max among participants in the Tabata training groups postintervention. However, the findings from Islam et al. Islam et al. (2020) presented a contrasting result. In their study, participants engaged in a 4-week intervention consisting of four weekly Tabata sessions, each comprising full-body functional exercises such as burpees, mountain climbers, jump squats, deep squats, and bench presses. Despite the adherence to the Tabata structure, no statistically significant improvements in ${\text{VO}}_{2}$ max were observed following the intervention. This discrepancy may be attributed to the short intervention period or to the participants’ superior baseline cardiorespiratory fitness levels. Indeed, prior research has indicated that baseline fitness status and initial training level can significantly influence training outcomes Milanović et al. (2015). Nevertheless, other studies have confirmed that Tabata training can effectively enhance aerobic and anaerobic capacity, which is consistent with the energy metabolism improvements observed in the present study Tabata et al. (1996); Sumpena and Sidik (2017).

In current sports training research, the benefits of high-intensity interval training (HIIT) have been widely recognized. Jiménez-Maldonado et al. (2018) demonstrated that HIIT can significantly enhance athletes’ energy metabolism and maximal oxygen uptake ( ${\text{VO}}_{2}$ max). Similarly, Helgerud et al. (2007) reported that HIIT improved metabolic function and reduced resting uric acid concentrations. Furthermore, Kistner et al. (2019) provided evidence that HIIT can enhance athletic performance by improving metabolic pathways. These findings align with the results of the present study, highlighting the broad impact of HIIT on cardiorespiratory function and energy metabolism. Although prior research has established the effectiveness of HIIT, most studies have primarily focused on lower-limb or whole-body exercises. In contrast, the present study, consistent with emerging evidence from other sport-specific contexts, emphasizes the unique benefits of HIIT for swimmers. Specifically, our investigation explores the impact of upper-limb-focused HIIT on the cardiorespiratory fitness and energy metabolism of competitive swimmers. This focus reveals the distinct value and potential application of targeted HIIT protocols in enhancing performance through sport-specific muscle group training.

These findings carry practical significance for competitive swim training. Incorporating upper-body HIIT can optimize training time and improve muscular strength and endurance, especially in muscle groups critical to performance. Coaches may consider adding upper-body HIIT to maximize both aerobic and anaerobic capacity, particularly in long-distance events where energy conservation is vital. This research focuses on upper-limb HIIT, a domain less explored in prior literature that has primarily emphasized cardiorespiratory endurance and lower-limb performance improvements Nugent et al. (2017); Pang (2022); Amara et al. (2023). By examining upper-body-specific adaptations, our findings contribute to a more nuanced understanding of HIIT’s modality-dependent effects. Furthermore, our results align with Hibbs et al. (2008), who highlighted the importance of muscle-specific training for sport-specific skill development—a principle we extend to upper-body resistance protocols.

Despite these promising results, limitations must be acknowledged. The relatively small sample size may limit the generalizability of the findings, and future research should involve larger and more diverse populations. The short intervention period also restricts long-term effect evaluation; hence, longer follow-up studies are warranted. Additionally, while this study focused on ${\text{VO}}_{2}$ max and energy metabolism, future investigations should explore other dimensions, such as muscular strength, endurance, technical improvements, and psychological factors like confidence and stress coping. Examining mental impact of upper-body HIIT may provide valuable perspectives for sports training science.

Future research should consider broader participant groups, diverse training models, and long-term tracking. Including swimmers of varying skill levels would yield more comprehensive insights into the effects of upper-body HIIT. Exploring variation in training modes, intensity, duration, and frequency may help determine optimal training protocols. Ultimately, long-term studies are essential for evaluating the sustained benefits of upper-body HIIT and for designing enduring, high-performance training plans.

## 5 Conclusion

This randomized controlled trial explored the effects of upper-body HIIT on energy metabolism and ${\text{VO}}_{2}$ max in swimmers. After a 4-week intervention, the HIIT group showed significantly greater improvements in energy system indicators and ${\text{VO}}_{2}$ max compared with the MICT group. These findings suggest that upper-body HIIT is an effective, time-efficient method for enhancing cardiopulmonary function and energy utilization in swimmers. For athletes seeking performance enhancement, upper-body HIIT can serve as a valuable addition to swim training programs. These findings demonstrate that upper-body HIIT enhances metabolic adaptations relevant to swimming physiology, including ${\text{VO}}_{2}$ max and substrate utilization efficiency. However, confirmation of practical application requires integration with kinematic analyses and performance trials because physiological improvements alone do not guarantee enhanced swimming outcomes. However, these results cannot be generalized to total-body HIIT because the distribution of workload, muscle mass recruitment, and systemic physiological responses likely differ substantially between upper-body-focused and total-body interventions. Future studies directly comparing upper-body, lower-body, and total-body HIIT designs would be valuable to clarify whether the observed effects are modality specific.

## Data Availability

The original contributions presented in the study are included in the article/supplementary material, further inquiries can be directed to the corresponding author.
